# Methyl eugenol regulates mating behavior in oriental fruit flies by enhancing lek attractiveness

**DOI:** 10.1093/nsr/nwae294

**Published:** 2024-08-22

**Authors:** Jie Zhang, Wei Liu, Hetan Chang, Qi Wang, Jinxi Yuan, Leyuan Liu, Chenhao Liu, Yan Zhang, Chuanjian Ru, Shanchun Yan, Bill S Hansson, Guirong Wang

**Affiliations:** Shenzhen Branch, Guangdong Laboratory of Lingnan Modern Agriculture, Key Laboratory of Synthetic Biology, Ministry of Agriculture and Rural Affairs, Agricultural Genomics Institute at Shenzhen, Chinese Academy of Agricultural Sciences, Shenzhen 518120, China; School of Forestry, and Key Laboratory of Sustainable Forest Ecosystem Management-Ministry of Education, Northeast Forestry University, Harbin 150040, China; Shenzhen Branch, Guangdong Laboratory of Lingnan Modern Agriculture, Key Laboratory of Synthetic Biology, Ministry of Agriculture and Rural Affairs, Agricultural Genomics Institute at Shenzhen, Chinese Academy of Agricultural Sciences, Shenzhen 518120, China; Shenzhen Branch, Guangdong Laboratory of Lingnan Modern Agriculture, Key Laboratory of Synthetic Biology, Ministry of Agriculture and Rural Affairs, Agricultural Genomics Institute at Shenzhen, Chinese Academy of Agricultural Sciences, Shenzhen 518120, China; Shenzhen Branch, Guangdong Laboratory of Lingnan Modern Agriculture, Key Laboratory of Synthetic Biology, Ministry of Agriculture and Rural Affairs, Agricultural Genomics Institute at Shenzhen, Chinese Academy of Agricultural Sciences, Shenzhen 518120, China; School of Forestry, and Key Laboratory of Sustainable Forest Ecosystem Management-Ministry of Education, Northeast Forestry University, Harbin 150040, China; Shenzhen Branch, Guangdong Laboratory of Lingnan Modern Agriculture, Key Laboratory of Synthetic Biology, Ministry of Agriculture and Rural Affairs, Agricultural Genomics Institute at Shenzhen, Chinese Academy of Agricultural Sciences, Shenzhen 518120, China; School of Forestry, and Key Laboratory of Sustainable Forest Ecosystem Management-Ministry of Education, Northeast Forestry University, Harbin 150040, China; College of Plant Health & Medicine, Qingdao Agricultural University, Qingdao 266071, China; Shenzhen Branch, Guangdong Laboratory of Lingnan Modern Agriculture, Key Laboratory of Synthetic Biology, Ministry of Agriculture and Rural Affairs, Agricultural Genomics Institute at Shenzhen, Chinese Academy of Agricultural Sciences, Shenzhen 518120, China; School of Forestry, and Key Laboratory of Sustainable Forest Ecosystem Management-Ministry of Education, Northeast Forestry University, Harbin 150040, China; Shenzhen Branch, Guangdong Laboratory of Lingnan Modern Agriculture, Key Laboratory of Synthetic Biology, Ministry of Agriculture and Rural Affairs, Agricultural Genomics Institute at Shenzhen, Chinese Academy of Agricultural Sciences, Shenzhen 518120, China; School of Forestry, and Key Laboratory of Sustainable Forest Ecosystem Management-Ministry of Education, Northeast Forestry University, Harbin 150040, China; Shenzhen Branch, Guangdong Laboratory of Lingnan Modern Agriculture, Key Laboratory of Synthetic Biology, Ministry of Agriculture and Rural Affairs, Agricultural Genomics Institute at Shenzhen, Chinese Academy of Agricultural Sciences, Shenzhen 518120, China; School of Forestry, and Key Laboratory of Sustainable Forest Ecosystem Management-Ministry of Education, Northeast Forestry University, Harbin 150040, China; School of Forestry, and Key Laboratory of Sustainable Forest Ecosystem Management-Ministry of Education, Northeast Forestry University, Harbin 150040, China; Department of Evolutionary Neuroethology, Max Planck Institute for Chemical Ecology, Jena 07745, Germany; Shenzhen Branch, Guangdong Laboratory of Lingnan Modern Agriculture, Key Laboratory of Synthetic Biology, Ministry of Agriculture and Rural Affairs, Agricultural Genomics Institute at Shenzhen, Chinese Academy of Agricultural Sciences, Shenzhen 518120, China; State Key Laboratory for Biology of Plant Diseases and Insect Pests, Institute of Plant Protection, Chinese Academy of Agricultural Sciences, Beijing 100193, China

**Keywords:** plant volatile compounds, oriental fruit fly, methyl eugenol, olfactory receptor

## Abstract

Plant-produced volatiles play a pivotal role as mediators in complex interactions between insects and plants. Despite the widespread recognition that these compounds serve as cues for herbivorous insects to locate their preferred host plants, their effects on insect mating behavior are less understood. Here, we show that male oriental fruit flies (*Bactrocera dorsalis*) are highly attracted to the host plant volatile compound methyl eugenol (ME), which enhances the attractiveness of male leks to females. To elucidate the molecular underpinnings of this phenomenon, we identify the olfactory receptor BdorOR94b1 responsible for detecting ME. Genetic disruption of *BdorOR94b1* leads to a complete abolition of both physiological and behavioral responses to ME. Additionally, we confirm that, through digestion, male flies convert ME to (E)-coniferyl alcohol, a compound that enhances the attractiveness of their leks to females. This increased attractiveness allows females to select optimal mates, thereby enhancing their reproductive success. The impairment of ME detection significantly diminishes the mating advantage within the leks, as males are unable to locate and utilize ME effectively. Our findings unveil a novel mechanism through which plant volatile compounds regulate the mating behavior of the economically important oriental fruit fly and provide new insights into the general ecology of insect–plant interactions.

## INTRODUCTION

Plant-produced volatile organic compounds (VOCs) serve as essential chemical signals facilitating communication between plants and insects, thereby playing pivotal roles in navigation, habitat location and inter-species interactions [1,2]. Particularly crucial is their function in guiding herbivorous insects to host plants, as plants emit specific volatile compounds that guide herbivores to locate suitable food sources [3]. This relationship not only satisfies the nutritional needs of herbivores but also shapes the dynamics of plant populations and herbivore communities within ecosystems [1,2,4]. Moreover, plant-produced VOC emission can be triggered by herbivore attack, activating plant defense mechanisms by attracting natural enemies like parasitoids and predators, thus bolstering plant survival [5–8]. Additionally, plant-produced VOCs contribute significantly to plant reproductive success by attracting pollinators, facilitating pollination and subsequent seed production [9–11]. This mutualistic association underscores the ecological importance of plant scents in modulating insect behavior and sustaining biodiversity within ecosystems.

In addition to their fundamental role in mediating plant–insect interactions, several studies have explored a nuanced aspect of plant-volatile-mediated insect behavior, particularly focusing on their influence on mating behavior. This influence can be exerted through various mechanisms. Firstly, the plant-produced VOCs can in some insects stimulate pheromone production, thereby amplifying mating behavior [12–14]. Secondly, plant volatiles can synergistically enhance the attractiveness of pheromones across diverse insect species, further promoting mating success [15–18]. Thirdly, plant volatiles can serve as long-range cues for mate location, particularly in parasitoids, aiding insects in finding potential mates [19,20]. Finally, components of plant volatiles can be ingested by insects and integrated into their pheromones, influencing mate attraction and reproductive success [21–25]. All these insights shed light on the intricate role of plant-produced VOCs in shaping insect mating behavior.

Methyl eugenol (ME) is a phenylpropanoid compound first identified from citronella oil for its attractiveness to tephritid flies [26,27]. Further field trap assays indicated that ME could attract various *Bactrocera* flies, particularly the oriental fruit fly, *B. dorsalis* [28–32]. ME has been characterized by its male-specific attraction and feeding, long-distance attractancy, effectiveness in minute quantities, and lack of repellency at high concentrations in the case of this pest [28,33]. These properties have led to the development of the well-known male annihilation technique (MAT) [34–42]. In nature, phenylpropanoids and phenylbutanones are key floral scents of *Bulbophyllum* orchids, essential for attracting specific *B.* fly species for one-to-one, species-specific pollination. Around 30 *Bu.* species depend entirely on these flies for pollination. *B. dorsalis* is notably attracted to and feeds on ME produced by the orchids *Bu. cheiri* and *Bu. vinaceum* [26,27,33,43–50]. This feeding behavior significantly boosts mating success [51–55]. Once consumed, ME undergoes transformation into structurally similar derivatives in the rectal gland of the male oriental fruit flies, believed to be part of its sex pheromone [21,23–25]. Male oriental fruit flies form multi-male leks, where female mate choice occurs [56]. It is possible that ME has a potential influence on the lekking behavior of *B. dorsalis*. However, further investigation is required to test this hypothesis and the underlying mechanisms still remain elusive.

In this study, we aimed to investigate the role of ME in the mating behavior of *B. dorsalis*. We found that male flies preferentially consume ME, leading to an increased production of (E)-coniferyl alcohol (ECF). This in turn enhances the attractiveness of male leks to females, allowing female mate-choice and reproduction. To elucidate the molecular basis underlying ME detection, we identified a type of olfactory sensory neuron responding to ME with high specificity and expressing the olfactory receptor BdorOR94b1. Genetic disruption of *BdorOR94b1* fully disrupted the fly response to ME. Our results thus unveil an alternative mechanism by which a plant odor can influence insect mating behavior.

## RESULTS

### Antennal olfactory receptor genes mediate the attraction response to methyl eugenol in *B. dorsalis*

**Figure 1.**
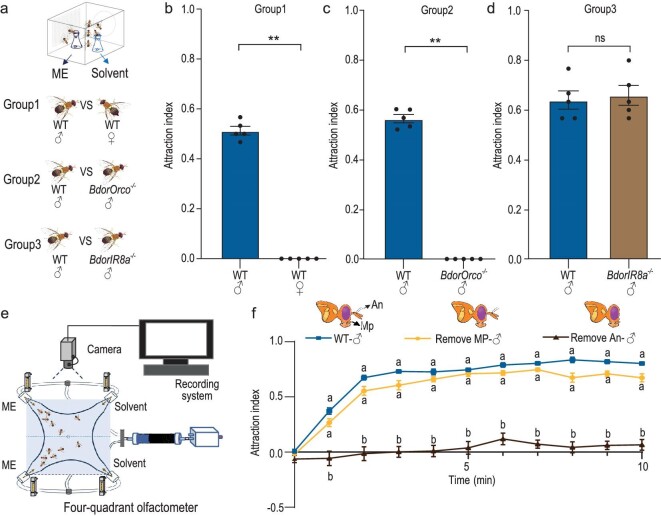
ME attraction in *B. dorsalis* males is mediated by olfactory receptors (ORs) expressed in antennal olfactory sensory neurons (OSNs). (a) Schematic drawing of the trap assay. (b) Comparison of ME trapping rates between WT males and females. (c) Comparison of ME trapping rates between WT and *BdorOrc*o^−/−^ males. (d) Comparison of ME trapping rates between WT and *BdorIR8a*^−/−^ males. Each experiment in (b), (c) and (d) comprised 5 replicates, with 30 individuals tested per replicate. Results are presented as the mean ± standard error. In (b) and (c), *P* values were conducted using a Wilcoxon rank-sum test, whereas *P* values in (d) were determined by a two-tailed unpaired t-test. (**P < 0.01; ns indicates no significant difference.) (e) Schematic drawing of the four-quadrant olfactometer assay. (f) Attraction index (AI) of intact males and males with removed antennae or maxillary palp. *n* = 5–7, with 30 individuals tested per replicate. Data are presented as mean ± standard error. The figure shows a comparative analysis of the differences in AI values among the three different males at each time point. For non-normally distributed data, the statistical analysis was conducted using the Kruskal-Wallis test followed by Dunn's multiple comparisons test, whereas normally distributed data were analyzed using one-way ANOVA followed by Tukey's multiple comparisons test (significant differences indicated by different letters, α = 0.05).

The oriental fruit fly, *B. dorsalis*, is attracted to ME [26,27], a primary floral metabolite in *B.* orchids. The flies feed in the orchid and simultaneously perform pollination services [45–47]. To assess the behavioral effects of ME on adult *B. dorsalis*, we conducted olfactory trap assays (Fig. S1a) and found a clear sexual dimorphism in response to ME, with males exhibiting a robust attraction to the compound, while females displayed no choice in comparison to a solvent control (Fig. 1a and b). Olfactory detection of volatile compounds primarily relies on two olfactory gene families in insects: olfactory receptors (ORs) and ionotropic receptors (IRs) [57–60]. To elucidate the specific roles of ORs in *B. dorsalis’* response to ME, we employed CRISPR-Cas9 genome editing to knock out *BdorOrco*, a co-receptor essential for the function of individual ORs (Fig. S1b–f). Our olfactory trap assays demonstrated that male *B. dorsalis* with a *BdorOrco* knockout completely lost their attraction to ME (Fig. 1c). This finding indicates that OR family genes are crucial for mediating the response to ME. In contrast, knock out of the ionotropic receptor *BdorIR8a* did not affect ME attraction (Fig. 1d, Fig. S1g–k), supporting the conclusion that ORs, rather than IRs, predominantly drive the attraction to ME. Given that insect OR genes are typically expressed in two olfactory organs, the antennae and the maxillary palps [61–63], we next sought to determine which olfactory organs were involved in the response to ME. We removed either the maxillary palps or antennae of male *B. dorsalis* and subjected them to a four-way olfactometer to test their behavioral response to ME (Fig. 1e, Fig. S1l). Removal of the maxillary palps from males had minimal effect on their attraction behavior towards ME, with only a slight decrease observed, possibly due to the minor trauma caused by dissection. In contrast, removal of the antennae completely eliminated the response (Fig. 1f), indicating that olfactory receptors expressed in antennal olfactory sensory neurons (OSNs) are crucial for the attraction of male *B. dorsalis* to ME.

### Identification of BdorOR94b1 as a specific receptor for ME detection

**Figure 2.**
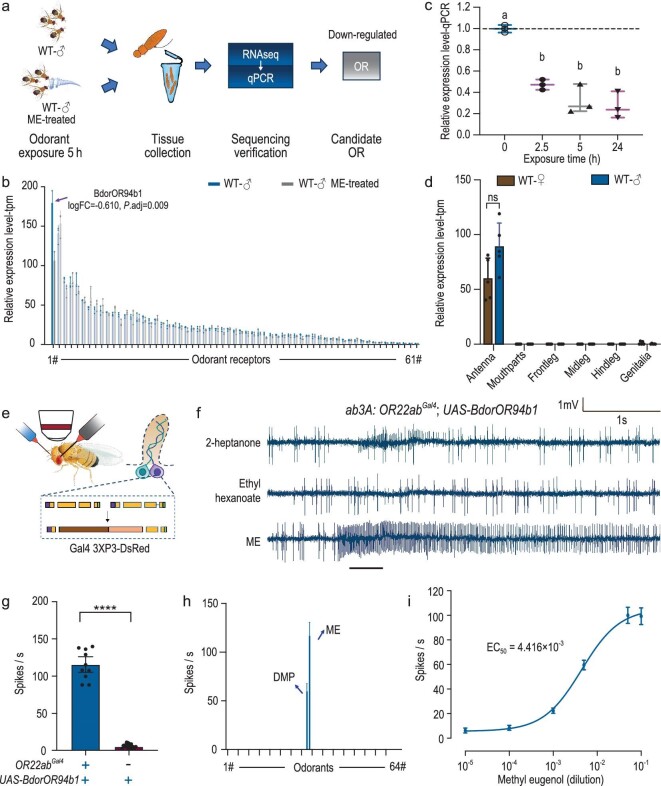
Identification of BdorOR94b1 as a specific receptor for ME detection. (a) Schematic drawing of the workflow for deorphanization of receptors based on the expression alterations in mRNA levels (DREAM) approach. (b) Comparison of the expression levels of antennal ORs before and after ME stimulation. (c) Relative expression level changes of *BdorOR94b1* in response to ME after different stimulation times. *n* = 3, data are presented as mean ± standard error. One-way ANOVA was used for data analysis, with Tukey's multiple comparisons test applied (significant differences indicated by different letters, α = 0.05). (d) Expression levels of *BdorOR94b1* in different olfactory organs and body parts of both male and female flies. *P* values were conducted using a two-tailed unpaired t-test (ns indicates no significant difference). (e) Schematics of the heterologous expression system using an empty *Drosophila* ab3A neuron. (f) Representative single-sensillum recording (SSR) traces from *Drosophila* ab3A neurons expressing *BdorOR94b1* responding to 2-heptanone, ethyl hexanoate and ME. (g) Quantification of ME responses in ab3A sensilla of *Drosophila* with or without expression of *BdorOR94b1*. ‘+’ and ‘-’ denote the presence or absence of *OR22ab^GAL4^* and *UAS-BdorOR94b1*, respectively. Results are expressed as mean ± standard error, with *n* = 10–14 for each group. *P* values were conducted using a two-tailed unpaired t-test (****P < 0.0001). (h) Tuning curves of BdorOR94b1 when stimulated with 64 compounds (Table S3), each compound applied at 100 μg. *n* = 9 recordings for each stimulation. (i) Dose-response curve of *BdorOR94b1* to ME. *n* = 9 recordings for each stimulation, data are presented as mean ± standard error.

To identify the specific *BdorOR* involved in detecting ME, we utilized the deorphanization of receptors based on expression alterations in mRNA levels (DREAM) approach [64,65], a method that takes advantage of the observation that mRNA levels of ORs change immediately after exposure to high concentrations of corresponding ligands (Fig. 2a, Fig. S2a–d). Our analysis revealed significant changes in gene expression within the antennae, with 436 genes being upregulated and 133 genes downregulated (Fig. S2c and d). We then analyzed the mRNA expression levels of *BdorORs* specifically. Among the olfactory receptors expressed in males, we identified *BdorOR94b1* as the only receptor exhibiting a significant reduction in expression following 5 hours of methyl eugenol (ME) exposure (Fig. 2b). Subsequent exposure of males to ME for durations ranging from 2.5 to 24 hours confirmed a sustained decrease in *BdorOR94b1* expression, consistent with the previous RNA sequencing findings (Fig. 2c). In addition, we validated the expression pattern of *BdorOR94b1* using the antennal neuronal transcriptome data set, which indicated that this receptor is specifically expressed in the adult antenna (Fig. 2d). To confirm BdorOR94b1 as an ME detector, we cloned and functionally expressed it in the ab3 empty neuron system of *Drosophila melanogaster*, lacking the endogenous receptor OR22a (Fig. 2e). Single-sensillum recording (SSR) measurements in the transgenic *D. melanogaster* lines revealed strong activation of BdorOR94b1 by ME when expressed in the ab3 sensilla (Fig. 2f and g, Fig. S2e). We then asked how specific BdorOR94b1 was to ME and screened 64 *B. dorsalis*-associated volatiles reported in previous studies [66] (Table S3). Remarkably, only ME and its analog, 2-allyl-4,5-dimethoxyphenol (DMP), elicited a response, with ME inducing the strongest response in a dose-dependent manner (Fig. 2h and i, Fig. S2f). We thus established BdorOR94b1 as a highly sensitive and specific detector of ME.

### Validating the essential role of *BdorOR94b1* in mediating ME-directed attractive behavior in male *B. dorsalis*

**Figure 3.**
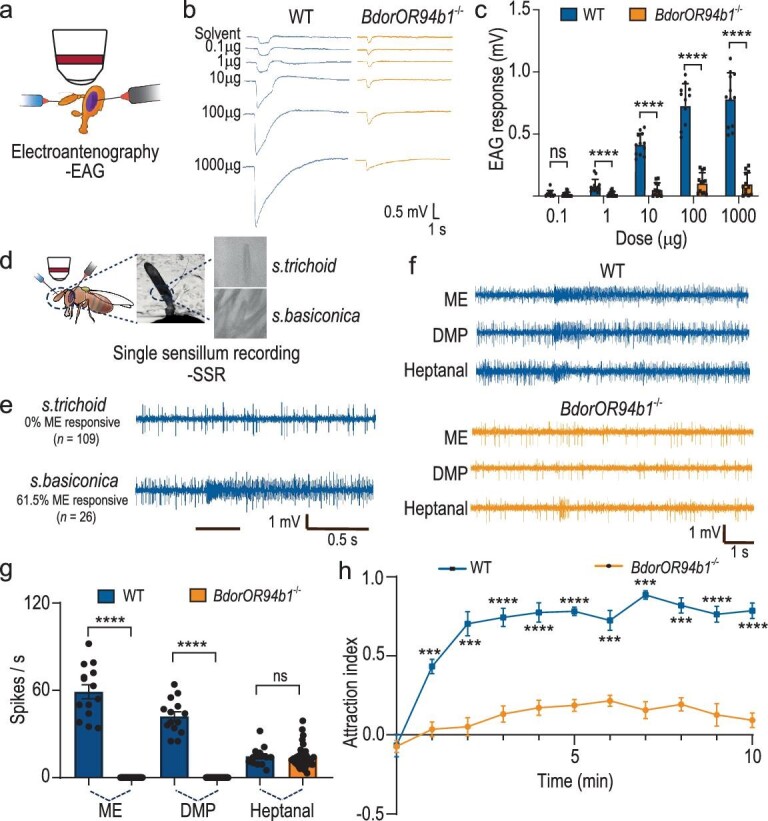
BdorOR94b1 is the key receptor that mediates ME-induced attraction behavior. (a) Schematic drawing of electroantennogram (EAG) experiments in *B. dorsalis*. (b) Representative EAG response traces from WT and *BdorOR94b1^−^^/^^−^* males stimulated by different concentrations of ME. (c) Quantification of EAG responses to different concentrations of ME in WT and *BdorOR94b1^−^^/^^−^* males, with *n* = 12 recordings in both types of males. (d) Schematic drawing of SSR experiments in *B. dorsalis.* (e) Representative SSR traces from *s. trichodea* (*n* = 109 recordings) and *s. basiconica* of *B. dorsalis* (*n* = 26 recordings). (f) Representative SSR response traces from *s. basiconica* in WT and *BdorOR94b1^−^^/^^−^* males stimulated with ME, DMP and heptanal. (g) Quantification of SSR responses to ME, DMP and heptanal by OSNs present in *s. basiconica* in WT and *BdorOR94b1^−^^/^^−^* males, with *n* = 14 sensilla (WT males) and *n* = 48 sensilla (*BdorOR94b1^−^^/^^−^* males). (h) Behavioral responses of WT and *BdorOR94b1^−^^/^^−^* males to ME. *n* = 7 biological replicates, each consisting of 30 individuals. All data in (c), (g) and (h) are presented as mean ± standard error. For non-normally distributed data, the statistical analysis was conducted using a Wilcoxon rank-sum test, whereas normally distributed data was analyzed using a two-tailed unpaired t-test (***P < 0.001, ****P < 0.0001, ns indicates no significant difference).

To confirm the crucial role of *BdorOR94b1* as the primary odorant receptor governing the response to ME, we utilized CRISPR-Cas9 genome editing to disrupt the gene encoding BdorOR94b1 (Fig. S3a and b). Employing a single guide RNA (sgRNA) targeting an exon of the *BdorOR94b1* gene, we induced a 124-base pair (bp) deletion, resulting in a truncated BdorOR94b1 protein (Fig. S3c–e). Subsequent examinations of the electrophysiological and behavioral responses of *B. dorsalis* to ME reaffirmed the significance of *BdorOR94b1* in mediating these responses. Electroantennographic (EAG) recordings revealed a complete loss of response to ME in the *BdorOR94b1*^−/−^ line compared to the wild type (WT) (Fig. 3a–c). Single sensillum recordings in WT *B. dorsalis* males confirmed that ME-responsive OSNs were located in *sensilla basiconica* (Fig. 3d and e). These OSNs responded exclusively to ME and DMP, with the strongest and dose-dependent response elicited by ME, consistent with findings from the *Drosophila* empty neuron system (Fig. S3f–i). When knocking out BdorOR94b1, the OSNs no longer responded to methyl eugenol (ME) and DMP. However, a residual response to heptanal was observed from another neuron within the same sensillum. The response to heptanal was comparable between WT and mutant flies (Fig. 3f and g). Finally, a four-quadrant olfactometer assay revealed an almost complete loss of attraction to ME in the *BdorOR94b1*^−/−^ line compared to the WT (Fig. 3h, Video S1 and 2). Collectively, our results from the CRISPR-Cas9-based experiments demonstrate that BdorOR94b1 is an indispensable and specific receptor governing ME-directed attractive behavior in male *B. dorsalis*.

### ME facilitates female localization of male leks in *B. dorsalis*

**Figure 4.**
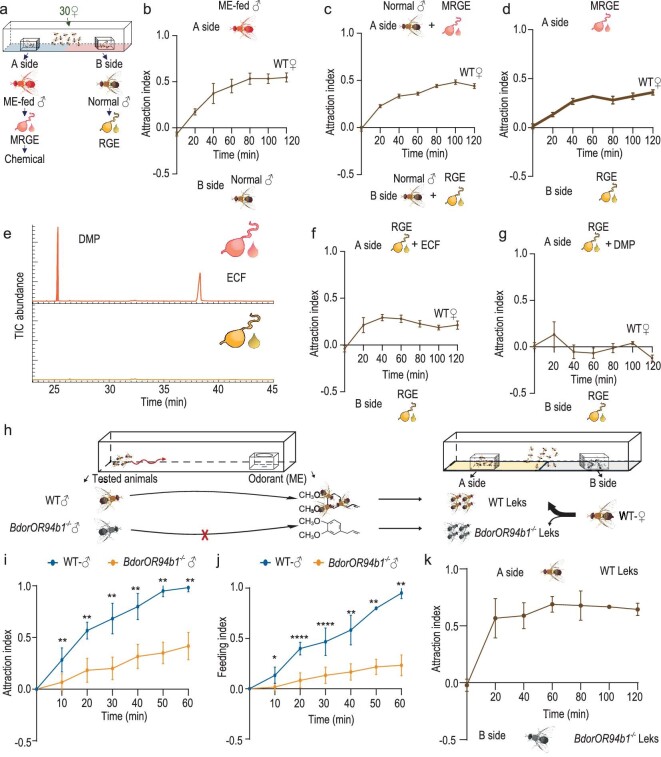
ME enhances female localization of male leks. (a) Schematic of dual-choice lek experiments. (b) Behavioral choice of WT female oriental fruit flies between male leks containing ME fed or untreated males, (c) between two leks, both containing untreated males, where one side had been supplemented with the odor of rectal gland extract from ME-fed males (MRGE), the other with extract from untreated males (RGE), (d) between empty lek cages supplemented with the MRGE and RGE. (e) Gas chromatograms showing the presence of the two main components in rectal gland extractions from ME-fed and untreated males. (f, g) The behavioral choice of WT female oriental flies between leks supplemented with untreated male rectal gland extract in combination with ECF as compared to pure gland extract (f), the same experiment repeated with supplement of DMP (g). (h) Schematic of the localization and feeding on ME by WT and *BdorOR94b1^−^^/^^−^* males, followed by the subsequent lek attraction assay to females after attraction and feeding assay. (i) Comparison of ME attraction between WT and *BdorOR94b1^−^^/^^−^* males. (j) Comparison of the feeding of WT and *BdorOR94b1^−^^/^^−^* males on ME. (k) Behavioral choice of WT female oriental flies between male leks containing WT and *BdorOR94b1^−^^/^^−^* males after attraction and feeding assay. In (b), (c), (d), (f) and (g), *n* = 5, each consisting of 30 individuals. In (i), (j) and (k), *n* = 6. Data are presented as mean ± standard error. In (i) and (j), for non-normally distributed data, the *P* values were analyzed using a Wilcoxon rank-sum test, whereas normally distributed data was analyzed using a two-tailed unpaired t-test (*P < 0.05, **P < 0.01, ****P < 0.0001).

In the field, the majority of *B. dorsalis* copulations typically occur within male lek formations [56], highlighting the significance of the female locating these lekking sites as the initial step in mating. We next aimed to investigate whether ME influences *B. dorsalis* mating behavior by facilitating female lek localization. To explore this, we provided two leks positioned on opposite sides of a rectangular cage, allowing females to choose (Fig. 4a, Fig. S4a–e). Initially, we compared female preference between a lek comprising males fed ME and another comprising untreated males. Exposed to this choice, females consistently favored the lek comprising ME-fed males over the untreated males (Fig. 4b). Considering the metabolic conversion of ME into analogs in the male rectal gland, alongside the release of sex pheromones during courtship [67], we next investigated whether rectal gland extract odors from ME-fed males would also attract females. We therefore presented two leks: one of the leks was supplemented with extract from the rectal glands of ME-fed males, while the other was supplemented with extract from the rectal glands of untreated males. Facing this choice, females exhibited a strong preference for the lek with rectal gland extract from ME-fed males (Fig. 4c). Consistently, females also favored the empty lek cage supplemented with rectal gland extract odors from ME-fed males over the cage supplemented with extract from the rectal glands of untreated males (Fig. 4d), underscoring the importance of compounds from ME-fed males as attractants for females. Previous studies have suggested that ECF and DMP are derivatives of ME [21]. Our findings confirm that ME is indeed converted into two prominent components stored in the male rectal gland: ECF and DMP (Fig. 4e, Fig. S4f–h). To test the hypothesis that these two compounds might facilitate the female localization of male leks, we supplemented leks with rectal gland extract from untreated males, adding either ECF or DMP to one of the extracts. Notably, only the cage where ECF had been added significantly attracted females (Fig. 4f and g). Furthermore, experiments involving female *B. dorsalis* lacking the odorant receptor co-receptor (*BdorOrco^−^^/-^* mutants) demonstrated a loss of preference for leks formed by ME-fed males (Fig. S4i) and ECF (Fig. S4j). These findings collectively suggest the critical role of ME-derived ECF in mediating female lek localization and that the process is mediated via olfaction. Due to the severe impact of *BdorOR94b1* deficiency on male flies’ ability to locate ME, we hypothesize that the loss of function of *BdorOR94b1* would attenuate their searching for and feeding on ME, thereby impairing ME-mediated lek enhancement. To test this hypothesis, we first conducted an attraction and feeding assay to evaluate the attraction and feeding rates of both *BdorOR94b1*^−/−^ and WT male flies towards ME. Subsequently, we assessed the attractiveness of the leks formed by these tested males to females within a rectangular cage over a 2-hour period (Fig. 4h). The results showed that the locating and feeding on ME of *BdorOR94b1*^−/−^ mutants were significantly reduced compared to WT males (Fig. 4i and j). As we expected, female flies showed a preference for leks formed by WT males over *BdorOR94b1*^−/−^ leks after the search and feed assay (Fig. 4k). This difference was not attributable to the mutant itself (Fig. S4k).

### The potential ecological significance of ME-directed lek location in female *B. dorsalis*

**Figure 5.**
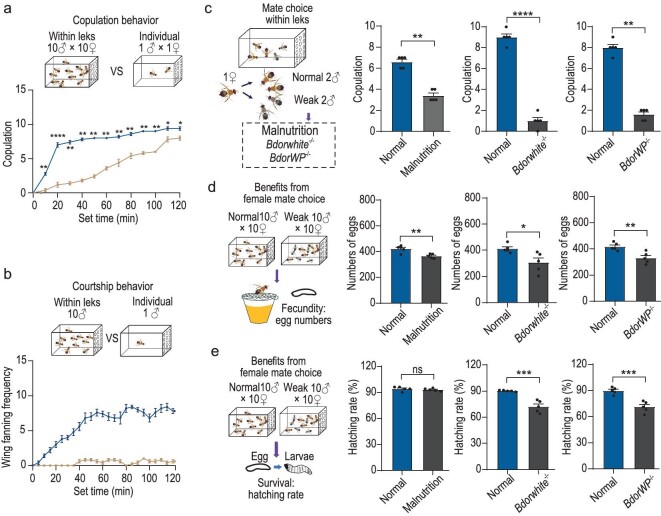
Female reproductive fitness benefits from mate selection within leks. (a) The copulation behavior in leks and in single pairs. The data represent the number of copulations observed during the experiment. (b) The courtship behavior exhibited by males within leks and individually. The data represent the number of flies that exhibited wing-fanning behavior during the observation period. (c) Mate choice of female oriental flies among two normal and two weak male flies. The weak males consisted of three types: malnourished, *Bdorwhite^−^^/^^−^* and *Bdorwp^−^^/^^−^*, all exhibiting impaired reproductive behavior to varying extents. (d, e) Comparison of egg production and egg hatching rates resulting from mating with normal and three types of weak males. In all experiments, *n* = 5. Data are presented as mean ± standard error. For non-normally distributed data, the *P* values were analyzed using a Wilcoxon rank-sum test, whereas normally distributed data was analyzed using a two-tailed unpaired t-test (*P < 0.05, **P < 0.01, ***P < 0.001, ****P < 0.0001, ns indicates no significant difference).

Since the majority of copulations (Fig. 5a) and preceding courtship behaviors in *B. dorsalis* (Fig. 5b) occur in leks rather than among solitary individuals, we next sought to determine whether females indeed select male sexual partners based on quality, thereby enhancing offspring quality and survival. To test this hypothesis, we conducted a series of competitive mating experiments, where we introduced two normal flies and two weaker ones (malnourished (Fig. S5a–e), *Bdorwhite^−^^/−^*[68] or *Bdorwp^−^^/−^*[66]) to a single female. We observed that female flies consistently preferred normal males over weaker ones in these miniature leks, regardless of the specific type of weakness (Fig. 5c). Subsequently, we evaluated the influence of female mate choice on fecundity (Fig. 5d) and observed that mating with weaker males resulted in a significant reduction not only in the number of eggs but also in larval hatching rates as compared to mating with normal males (Fig. 5e). Leks could thus play a crucial role in attracting females to an arena, where male quality can be directly assessed before copulation, ultimately contributing to enhanced offspring quality and survival.

## DISCUSSION

Lekking is a widespread phenomenon observed across various taxa, spanning mammals, anurans, fish and insects [69]. Among tephritid flies, an example of lekking insects, several species have been investigated and shown to display male leks. These species include *B. cucurbitae* [70], *B. oleae* [71], *Ceratitis capitata* [72], *Anastrepha luden*s [73] and *Rhagoletis pomonella* [74]. The lek formation emits a multifaceted and multisensory signal consisting of visual, acoustic and olfactory cues, with olfactory cues, particularly the long-range pheromones emitted by lekking males, attracting females to sites of behavioral display [75,76]. Our study reveals that male oriental fruit flies are highly attracted to the host plant volatile compound ME, produced by *Bu.* orchids. Male flies convert ME to ECF, which enhances the attractiveness of male leks to females. Observations of the mating system in a closely related species, *B. cacuminata*, near artificial ME sources [32], suggest that lek formation may rely on ME, with males aggregating around natural ME sources such as *Bu.* orchids. These orchids serve as rendezvous sites where pheromones produced from the rectal gland, such as ECF or pyrazines [67], combine with acoustic signals to attract females and facilitate optimal mate choice.

Moreover, we reveal the molecular mechanisms underlying the male *B. dorsalis* attraction to ME. One specific OR, BdorOR94b1, is underpinning the behavioral response to ME. Previous studies demonstrated *BdorOR88a* as another OR detecting ME [77]. The *B. dorsalis* T2T genome in turn revealed three copies of *BdorOR88a* arranged as tandem repeats. Even though a knockout of the highly expressed *BdorOR88a1* and *BdorOR88a2* resulted in attenuated responses to ME, the electrophysiological response of the antennae seemed to remain unaffected (Liu *et al.*, unpublished data). However, the knockout of *BdorOR94b1* in our current study almost abolished both the antennal electrophysiological response and the searching behavior for ME in male *B. dorsalis*, highlighting this receptor's significance in allowing olfactory behavior towards ME. Detection of one specific odor by multiple ORs has been observed in several insect species, also in some cases relevant for the detection of pheromones. In *D. melanogaster*, the pheromone compound cVA is detected by multiple ORs, each mediating specific behaviors to different concentrations of the pheromone [78]. Thus, the complicated receptor system underlying the detection of ME in *B. dorsalis* might also allow e.g. concentration-dependent modifications to behavioral responses.

Our findings reinforce the idea that central nervous system processing plays a crucial role in determining the behavioral responses to odors in insects, beyond the expression of ORs. In *D. melanogaster*, the sexually dimorphic central neural pathways associated with the odorant receptor DmelOR67d, which detects cVA, illustrate how OSNs and their projection neurons shape behavior [79,80]. Similarly, in the oriental fruit fly, both males and females use the receptor BdorOR94b1 to detect ME. This implies that the neural circuits involved in processing ME may also exhibit sexual dimorphism. Given that ME is currently used predominantly for managing male adults, exploring the function of BdorOR94b1 in females could provide valuable insights into expanding pest management strategies to target both sexes.

The adult male of *B. dorsalis* is highly attracted to ME and will consume plant products containing it directly upon location [28,33]. ME is then rapidly metabolized and converted into various ME derivatives before being stored in the rectal gland, with ECF and DMP being the predominant byproducts [21]. Both ECF and DMP attract females, with ECF being more potent [81,82]. Our results show that the presence of males that have ingested ME increases the attractiveness of leks to females, and that ECF from the male rectal gland is the key component driving this effect. A direct effect of ingested precursors to pheromones has been found in other insects. In the moth *Grapholita molesta*, males that were fed on the natural food choice, apple, produced significantly higher amounts of the male pheromone ethyl trans-cinnamate. Sequestration of this compound into the hair pencils also increased male mating success [83]. In another system, males of the African monarch butterfly, *Danaus chryshippus*, produce their pyrrolizidinone pheromone only after consuming precursors from *Heliotropium* species. The food plant of this species is, however, the *Asclepia* species, while it seems that the *Heliotropium* is visited by males exclusively to obtain the pheromone precursor lycopsamine [84]. These examples from other insects illustrate how the ingestion and conversion of chemical compounds might enhance attractiveness, probably similar to the role of ECF. In the case of ECF in the female oriental fruit fly, the knockout of the olfactory co-receptor *BdorOrco* completely eliminated this enhanced attractiveness, showing that OR-based olfaction regulates female attraction to the leks. Given that BdorOR94b1 does not react to ECF, a different OR might be involved in female detection of ECF. Further studies are needed to allow the identification of the female OR or ORs involved in the detection of ECF.

In conclusion, the impact of ME on the mating behavior of the oriental fruit fly was elucidated through a combination of functional genomics, electrophysiology and behavioral analyses. Our findings not only provide new insights into the ecology of insect–plant interactions but also have practical implications for the development of pest management strategies using phenylpropanoids as behavior-modifying agents for insects.

## Supplementary Material

nwae294_Supplemental_File
