# Supplementary material for: Methyl eugenol regulates mating behavior in oriental fruit flies by enhancing lek attractiveness
Source: Natl Sci Rev. 2024 Aug 22;12(3):nwae294. doi: 10.1093/nsr/nwae294 (PMC11846082; doi:10.1093/nsr/nwae294)
Supplement: nwae294_Supplemental_File [file nwae294_supplemental_file.pdf]

Supplemental Information for

Methyl eugenol regulates mating behavior in oriental fruit flies by enhancing lek attractiveness

Authors: Jie Zhang[1,2†], Wei Liu[1†], Hetan Chang[1†], Qi Wang[1,2],Jinxi Yuan[1,2], Leyuan Liu[3], Chenhao Liu[1,2], Yan Zhang[1,2], Chuanjian Ru[1,2], Shanchun Yan[2], Bill S Hansson[4], Guirong Wang[1,5*]

This PDF file includes:

METHODS

Figs. S1 to S5

METHODS

Animals

The lines of *B.dorsalis* in Agricultural Genomics Institute at Shenzhen was a well-established laboratory strain originally from south China Agricultural University. They were kept under a 14-hour light to 10-hour dark photoperiod, at a temperature of 26±1°C and a relative humidity of 60±5%. The larvae were provided with an artificial diet composed of banana, corn flour, yeast, sugar, and cellulose paper. When the larvae reached maturity, they were collected and placed in moist sand to pupate. After pupation, the emerging adults were separated by sex within 3 days and housed in small cages (18 cm × 12.5 cm × 14 cm). Each cage contained 40 adults and was supplied with water and a yeast-sugar mixture in a 1:1 ratio for nourishment.

Odorant exposure, RNAseq and qPCRs

The exposure procedure was adapted from the published work on mice and *Drosophila* with some modifications [1]. In summary, the odorant exposure experiment was conducted in a small cage (18 cm × 12.5 cm × 14 cm). The insects used in the experiment were 12-day-old, unmated, sexually mature males. Initially, the males were kept in a culture room free from any external odor interference, with access only to adequate diets and water. Both the ME treatment group and the control group consisted of 100 males each. During the exposure experiment, the 100 males were divided into two smaller cages, with 50 males in each cage, to ensure complete exposure to ME. In the exposure cage, a clean filter paper infused with 20 μl of ME (100 μg/μl, dissolved in paraffin oil) was placed, while a filter paper with only paraffin oil was placed in the non-exposure cage as a control. The antennae were collected immediately after 5 hours of exposure (from 9:00 to 14:00), flash-frozen in liquid nitrogen, and stored at -80°C for subsequent RNA extraction and transcriptome sequencing. Both the treatment and control groups included three replicates (each consists of 100 individuals).

RNA sequencing was conducted on the Illumina NovaSeq6000 sequencing platform. The raw data were processed using Trimmomatic software (version 0.39) to remove adapter sequences and low-quality reads, defined as those with a quality score below 20 or a sequence length shorter than 40 bp, thereby obtaining high-quality sequencing data. The high-quality data were then aligned to the reference genome (China National GeneBank Database: PRJCA020830), which had been previously assembled and annotated, using the HISAT2 software (version 2.2.1). The software featureCounts (version 2.0.1) was employed to quantify the expression levels of odorant receptor genes, resulting in the creation of their raw expression matrix, FPKM matrix, and TPM matrix. First, a box plot of the FPKM value matrix was generated using the ggplot2 package (version 3.4.2) to evaluate the similarity of expression patterns among samples. Second, hierarchical clustering of the different samples was conducted based on their FPKM values using the hclust function in R, and the correlation between samples was assessed based on the distance between them. Third, the correlation coefficient between samples was calculated using the cor function in R, based on the FPKM value matrix, and the strength of the correlation was determined by the magnitude of the correlation coefficient. Finally, principal component analysis (PCA) on the FPKM value matrix was performed using the FactoMineR (version 2.8) and factoextra (version 1.0.7) packages to identify differences between the ME treatment group and the control group. Differential expression analysis between these two groups was carried out using the DESeq2 package (version 1.40.1). The criteria for identifying differentially expressed genes (DEGs) were

set at an adjusted P-value (P.adj) of less than 0.05 and an absolute log2 fold change (|log2FC|) greater than 1.5. All analyses were conducted in R studio version 2023.09.1, which is built on R version 4.3.2. The relative expression levels of all genes are represented using TPM values. For the expression pattern of certain olfactory receptors in peripheral nervous tissues, the existing transcriptome data (antenna, mouthparts, front leg, mid leg, hind leg, genitalia) for the *B. dorsalis* genome assembly were directly used (China National GeneBank Database: PRJCA020830).

Quantitative real-time PCR (qRT-PCR) was employed to validate the differential expression of key odorant receptors identified through transcriptome analysis. Male insects were exposed to ME for various durations (2.5h, 5h, 24h), and the procedures for stimulation and tissue collection were consistent with the previously described protocol for the stimulation transcriptome. Total RNA was extracted from the antennae of the different stimulation groups using TriZol reagent (Invitrogen, Carlsbad, CA, USA). Approximately 1 μg of total RNA was used as a template for cDNA synthesis, which was conducted using the HiScript® III 1st Strand cDNA Synthesis (+gDNA wiper) Kit (Vazyme, Nanjing, China). The synthesized cDNA served as the template for qRT-PCR and was preserved at -20°C. Specific primers targeting the *BdorOR94b1* gene sequence, as determined by genome analysis, were designed using the NCBI Primer-BLAST tool (https://www.ncbi.nlm.nih.gov/tools/primer-blast/index.cgi?LINK_LOC=BlastHome). The α-tubulin gene served as the internal reference [2]. The PCR reaction mixture totaled 10 μL, comprising 5 μL of 2× Taq Pro Universal SYBR qPCR Master Mix (Vazyme, Nanjing, China), 0.5 μL of each primer (10 μM), 1 μL of cDNA, and 3 μL of RNase-free water. PCR amplification was conducted on a CFX96 real-time PCR detection system (Bio-Rad, Hercules, CA, USA). The primer sequences and PCR conditions are provided in Supplementary Table S1. Dissociation curve analysis was performed to confirm the specificity of the amplification prior to the main experiments. Each of the 0, 2.5, 5, and 24-hour exposure durations were tested for three biological replicates (a single replicate contains antennae from 50 individuals). The relative expression levels of each gene were calculated using the $2^{-\Delta\Delta CT}$ method [3].

Establishment of UAS lines in transgenic *Drosophila*

Total RNA was extracted from the antennae of male insects using TriZol reagent (Invitrogen, Carlsbad, CA, USA). The quality of the extracted RNA was evaluated using a NanoDrop ND-1000 spectrophotometer and by gel electrophoresis. High-quality RNA was subsequently treated with DNase I to remove any contaminating DNA, and then reverse-transcribed using the RevertAid First Strand cDNA Synthesis Kit (Thermo Scientific) to synthesize cDNA. This cDNA was diluted to a concentration of approximately 300 ng/μL and stored at -20°C to be used as a template for subsequent cloning procedures.

The specific primers for *BdorOR94b1* were used for amplification under the following conditions: initial denaturation at 98°C for 3 minutes; followed by 35 cycles of denaturation at 98°C for 10 seconds, annealing at 60°C for 15 seconds, and extension at 72°C for 1 minute and 30 seconds; with a final extension step at 72°C for 10 minutes; the samples were then held at 10°C. After purification, the PCR products were cloned into the pUAST-attB vector, and the recombinant plasmids were extracted and purified using the Qiagen midiprep kit. These plasmids were injected into the embryos of *D. melanogaster* (genotype: *y[1]M{vas-int.Dm}ZH-2A w[*]*;*P{CaryP}attP2*) (Fly microinjection was conducted by the Core Facility of Drosophila Resource and Technology, Center for Excellence in Molecular Cell Science, CAS). The G0

generation of *Drosophila* was crossed with a balancer line (genotype: *W*; *sp/Cyo*; *TM2/TM6B*), resulting in the acquisition of a UAS effector line with the genotype *W; sp/Cyo*; *UAS-BdorOR94b1/TM2*. This effector line was then crossed with the Or22a^{-}b^{-}Gal4 line (genotype: *W*; *22abGAL4*/*22abGAL4*; *TM2/TM6B*). The homozygous Gal4-UAS lines (genotype: *W*; *22abGAL4/22abGAL4*; *UAS-BdorOR94b1*/*UAS-BdorOR94b1*) were ultimately used for single sensillum recordings.

Establishment of the olfactory receptor mutant lines using CRISPR-Cas9

The establishment of olfactory receptor knockout mutants was conducted following the methods previously established by Yuan et al. [4]. This procedure included the synthesis of sgRNA, injection into embryos, and screening for mutants. For *BdorOrco*$^{-/-}$, *BdorIR8a*$^{-/-}$, and *BdorOR94b1*$^{-/-}$ mutants, the establishment of these mutants was done in this study. The *Bdorwhite*$^{-/-}$ and *Bdorwp*$^{-/-}$ mutants were directly used from mutants established in previous published lab studies.

The full-length structures of *BdorOrco*, *BdorIR8a*, and *BdorOR94b1* were predicted using the previously assembled genome of *B. dorsalis*, which is available in the China National GeneBank Database (Project ID: PRJCA020830). Genomic DNA was extracted from the mid-legs of adult flies, and the regions corresponding to the aforementioned genes were amplified via PCR with specific primers (details of the primers and PCR conditions are provided in Supplementary Table S1). The PCR products for each gene were cloned into blunt-end vectors (TransGen Biotech, Beijing, China). Subsequently, 20 individual bacterial colonies for each gene product were screened to identify conserved regions within the genes for the purpose of designing targets for gene editing. Utilizing the sgRNA-Cas9-AI software [5], we designed sgRNA targeting sites that were 20 base pairs in length and included an adjacent protospacer adjacent motif (PAM) of NGG or CCN (see Supplementary Table S1 for details). To enhance the probability of inducing mutations, more than two sgRNAs were designed for each gene. The sgRNAs were synthesized using commercial kits (GeneArt gRNA Kit, Thermo Fisher Scientific).

Adults aged 9-12 days were kept in transparent cages with sufficient food and water provided. Three days were allowed for mating to occur, after which an embryo collection apparatus containing orange juice was introduced. Embryos collected within a 10-minute window were used for injections. The working concentrations were set at 300 ng/μL for sgRNA and 150 ng/μL for Cas9 protein. After preparing the mixture of these components, injections into the embryos were performed using the FemtoJet and InjectMan 4 systems (Eppendorf, Hamburg, Germany). The injected embryos were subsequently incubated at 26.5°C with 60% relative humidity. Larvae that emerged were gently transferred with a soft brush to artificial diets and reared under the laboratory conditions.

Genomic DNA was extracted from the mid-legs of mutant adult flies for genotyping, with the specific primers and PCR conditions provided in Supplementary Table S1. The PCR-amplified fragments were analyzed by Sanger sequencing, and those individuals showing distinct overlapping peaks near the target site were identified as mutants carrying gene-editing events. The PCR products were then cloned into blunt-end vectors for precise genotype determination, selectively retaining only those strains with base deletions that led to frame shifts and premature stop codons. Individual adult G0 flies were backcrossed with wild-type flies, and genotyping was performed on the G1 offspring to isolate heterozygous mutants. The G1 heterozygotes with the

desired mutation were selected and crossed with wild-type adults to expand the population and generate multiple G2 heterozygotes. These G2 heterozygotes were intercrossed to yield G3 homozygotes. The G3 homozygous lines were established and maintained for subsequent experiments. Further details on the screening of knockout mutant lines can be found in Supplementary Table S2.

Identification of Chemical Compounds

Adult male flies, 12 days old and sexually mature, were used in this experiment. They were segregated into two groups, each consisting of 60 individuals. The treatment group was administered 20 μl of methyl eugenol (ME, 100 μg/μl in paraffin oil), whereas the control group received paraffin oil alone. The insects were allowed to feed for 8 hours (09:00-17:00), after which they were maintained under standard conditions until 17:00 the following day. At this time, the rectal glands were excised and immediately preserved in 1 ml of methanol. After 24 hours of extraction, the samples were filtered through a 0.22 μm nylon filter. The resulting filtrate was stored in 2 ml amber vials at -20°C until analysis.

The methanol extraction of rectal glands was analyzed using GC-MS (TSQ9000-Trace1310, Thermo Fisher Scientific, USA) with a DB-WAX UI column (30 m × 0.25 mm × 0.25 μm, Agilent, USA). The temperature program involved an initial column temperature of 50°C, held for 2 minutes, followed by an increase of 10°C/min to 230°C, which was then maintained for an additional 40 minutes. The injector temperature was set to 250°C, with an injection volume of 1 μL, using helium as the carrier gas in a non-split mode at a flow rate of 1.2 mL/min. The mass spectrometry parameters were as follows: electron impact (EI) ionization source temperature at 300°C, electron energy at 70 eV, and a mass range scan from 41 to 400 m/z. The compounds in the methanol-preserved rectal gland samples were initially identified through the utilization of the computer-aided NIST mass spectral library and manual verification. Subsequently, based on the preliminary identification results, corresponding standards were synthesized and reinjected for confirmation. Compounds that matched the standards were considered the final identification results.

Behavior Tests

The test insects were unmated adults (sorted by sex within three days post-emergence), 12 days post-emergence, and were housed in small insect cages (18 cm × 12.5 cm × 14 cm) prior to the experiments. Each cage accommodated 40-45 adults, and ample food and water were provided. After reaching maturity, healthy and active adults were selected for the behavioral experiments.

Olfactory trap assay: the two-choice olfactory trap assay was performed as previously described [4]. The trap was a clear conical flask with a top diameter of 3 cm, a base diameter of 5 cm, and a height of 7.5 cm. It featured an entry point for adult insects, which was created using the tip of a 1 mL pipette attached to the flask. Once the adult insects entered the trap, they were unable to escape. These traps were placed inside a large insect cage (24 cm × 18 cm × 14 cm), with two traps set in each cage. The first trap, designated as the experimental group, was baited with the test odorant ME, which was diluted in paraffin oil to a concentration of 100 μg/L and applied in a volume of 10 μL. The second trap served as the control and contained only paraffin oil. The experiments were conducted from 9:00 to 10:00 AM under a consistent light intensity of 280-300 lux (LED light pad with 20-50hz flicker fusion frequency). The temperature and relative humidity

of the experimental environment were maintained at 26 ± 1°C and 60 ± 5%, respectively. At the end of the experiment, the number of adults captured in each trap was counted. The attraction index was calculated as follows: (numbers of adults in treatment (for example, ME) trap-numbers of adults in control (for example, paraffin oil) trap)/numbers of adults tested. The experiment was replicated five times, with each replicate involving 30 individuals.

Four-quadrant olfactometer assay: the behavioral experiment was conducted using a four-quadrant olfactometer and was recorded by the laboratory's automated camera system. Before the experiment began, the olfactometer was thoroughly cleaned (wiped with 75% ethanol and dried for 5 minutes, repeated three times), and the stability of the gas flow was pre-tested to ensure that the flow rate in all four quadrants remained stable at 0.4 L/min. During the behavioral assay, the adult males being tested were introduced into the four-quadrant olfactometer, which was then well-ventilated. Time was allowed for the insects to acclimate to the airflow and to distribute themselves evenly across the quadrants. ME at a concentration of 1 μg/L and a volume of 10 μL dissolved in paraffin oil, was added to the glass odor chambers. The odor sources were connected to two quadrants on one side, while the control substances were connected to the two opposite quadrants. The response behavior of the adults was monitored for 10 minutes following the introduction of the odorant. The experimental conditions were maintained with a light intensity of 280-300 lux (LED light pad with 20-50hz flicker fusion frequency), a temperature of 26 ± 1°C, and a relative humidity of 60 ± 5%. The experiments were conducted between 8:00 and 9:00 AM. After the experiment, the videos were analyzed manually, and the data were collected. The number of adults in the four quadrants of olfactometer was counted every 1 minute. The attraction index was calculated as follows: (numbers of adults in test quadrants-numbers of adults in control quadrants/numbers of adults in test quadrants+numbers of adults in control quadrants. The experiment was replicated five times, with each replicate consisting of 30 adults. The males with maxillary palps or antennae removed were prepared one day in advance. The males were anesthetized on ice, and then the antennae and maxillary palps were gently removed with fine tweezers and scissors. After the removal procedure, these males were kept under normal rearing conditions until tested.

Lek attraction assay: the ME feeding protocol for the male insects was carried out according to the procedures described in the stimulation transcriptome. After feeding, the males were kept under the laboratory conditions, with adequate food and water supplied. The following day at 5:00 PM, the rectal glands were excised from some of the males and immersed in 1 ml of methanol, while the others were set aside for preparation for the behavioral experiment. The glands were allowed to soak in the methanol for a 24-hour period. Subsequently, any impurities were filtered out, and the solution was then concentrated to approximately 500 μl. Each extract contained the concentrated methanol solution from the rectal glands of 60 individuals.

The experimental setup consisted of a rectangular mesh cage (90 cm × 30 cm × 35 cm), with two smaller insect cages (18 cm × 12.5 cm × 14 cm) placed on each side. One side of these smaller cages was designed with a dense array of small holes to allow air to enter. At 16:30-18:30, ten males were introduced into each of the smaller cages to form leks under experimental conditions that included a light intensity of 50-80 lux [6], a temperature of 26 ± 1°C, and a relative humidity of 60 ± 5%. A total of 50 μL of rectal gland extract was evenly applied to the side of the cage with the dense array of small holes. The experimental females were 12-day-old unmated individuals that had not undergone any prior treatment. During the experiment, 30 female insects

were randomly released into the larger cage to ensure an even distribution. The two smaller cages, containing male leks or rectal gland extracts subjected to different treatments, were situated on opposite sides of the larger rectangular mesh cage. The females were allotted a period of 2 hours to make their choice between the male leks or the rectal gland extracts. Female preference was evaluated by calculating an attraction index (AI), which was determined by their distribution between the two sides of the rectangular mesh cage, using the formula AI = (NA - NB) / (NA + NB), where NA is the number of females on side A, and NB is the number of females on side B. An AI value close to zero would indicate no preference. This distribution data was recorded at 20-minute intervals, and a total of five replicates were conducted for each experimental condition. The various combinations of male leks or rectal gland extracts are detailed in the subsequent sections.

ME-Fed leks: These leks consist of 10 males that have been fed ME. Normal leks (as control): These leks are composed of 10 males that have not been fed ME.

Normal leks + ME-Fed Rectal Gland Extract: These leks include 10 males not fed ME, but the cage contains rectal gland extract from males that have been fed ME. Normal leks + Normal Rectal Gland Extract (as control): These leks comprise 10 males not fed ME, along with rectal gland extract from males that also have not been fed ME.

Rectal Gland Extract from ME-Fed Males: This setup features only the rectal gland extract from males that have been fed ME, without any males present. Normal Rectal Gland Extract (as control): This arrangement includes only the rectal gland extract from males that have not been fed ME, with no males present.

Normal Rectal Gland Extract ECF: This configuration contains the rectal gland extract from males not fed ME, supplemented with 5μl of ECF (at a concentration of 100μg/μl). Normal Rectal Gland Extract + 2-ally-4,5-dimethoxyphenol (DMP): In this setup, the cage includes rectal gland extract from males not fed ME, along with 5μl of DMP (at a concentration of 100μg/μl). Normal Rectal Gland Extract (as control): This arrangement includes only the rectal gland extract from males that have not been fed ME, with no males present.

Lek Attraction Assay to Females After Attraction and Feeding Assay: For the localization and feeding assay, the experimental setup consisted of a rectangular mesh cage (90 cm × 30 cm × 35 cm), with a smaller insect cage (18 cm × 12.5 cm × 14 cm) containing an opening (10 cm in diameter) placed on one side (Fig. 4h). We placed 10 μl of ME (at a concentration of 10 μg/μl) on one side of the small rectangular cage and released 10 male insects (either BdorOR94b1$^{-/-}$ or wild-type male flies) on the opposite side. Using the center of the cage as a boundary, we recorded the attraction index (AI) of the males to ME over a one-hour period and measured the feeding index (FI) on ME. The attraction index (AI) was determined by the number of males attracted to side ME of the rectangular mesh cage, using the formula AI = N(ME) / 10, where N(ME) is the number of males on side ME. The feeding index (FI) was determined by the number of males feeding on ME, using the formula FI = N(FME) / 10, where N(FME) is the number of males feeding on ME. After one hour, all the male insects were collected into a rearing cage using a small tube. These males were then used in a lek attraction assay with females at nightfall the following day. For the lek attraction assay to females, the apparatus and methods used were the same as those described in the previous lek attraction experiment. We assessed the attractiveness of the leks formed by these tested males to females.

Copulation assay: the copulatory behavior of males towards females was observed in a transparent cage (18 cm × 12.5 cm × 14 cm) under environmental conditions that included a light intensity of 50-80 lux, a temperature of 26 ± 1°C, and a relative humidity of 60 ± 5%. These observations took place between 16:30 and 18:30. The characteristic copulatory behavior is described as follows: At dusk, when a female approaches, the male leaps onto the dorsal abdomen of the female, flaps its wings, and initiates copulation. A successful copulation typically lasts for more than two hours [6]. Both the male and female adults used in the study were 12-day-old unmated individuals, and each trial was replicated 5 times.

To assess the differences in sexual behavior with and without the presence of leks, both group copulation trials and one-on-one copulation trials were conducted. In the group mating trials, 20 adults with a 1:1 male-to-female ratio were placed in the cage. In contrast, the one-on-one mating trials involved the observation of just a single male and female pair. The mating pairs among the adults were manually recorded every 10 minutes.

To evaluate whether females selectively chose males within leks, competition copulation trials were conducted. Each replicate included 10 such competition experiments, as described below. In each experiment, 5 individuals were placed in the same cage, where a female was given the option to choose between normal males (2 individuals) and less competitive males (2 individuals). Three types of less competitive males were utilized: malnourished males, which resulted from nutritional deprivation during the third day after larval hatching; and *Bdorwhite*$^{-/-}$ and *Bdorwp*$^{-/-}$ males, which exhibited impaired reproductive behavior to some degree [7, 8]. The number of mating pairs was manually tallied at the conclusion of the experiment.

Frequency of wing fanning: The frequency of wing fanning behavior is a typical courtship behavior of the oriental fruit fly [9], which was observed under the same conditions as those described in copulation assay section. Male adults that exhibited wing vibrations accompanied by abdominal quivering were identified as displaying typical wing fanning behavior. These behaviors were recorded using an automated video system and analyzed using the method previously described by Ren et al. [6]. To determine the difference in the frequency of wing fanning with and without the presence of leks, both group and individual wing fanning trials were conducted. In the group wing fanning trials, 10 males were placed in the cage to encourage lek formation. In contrast, the individual wing fanning trials involved observing only a single male in the cage (10 cages). Data were recorded at 5-minute intervals, and each trial was repeated five times.

Oviposition assay: To assess whether females could benefit from mate selection within the lek, we compared the fecundity of females after mating with normal males and with less competitive males (malnourished, *Bdorwhite*$^{-/-}$, and *Bdorwp*$^{-/-}$ males). The basic conditions were the same as those described in frequency of wing fanning section. In summary, a single replicate consists of 20 individuals (10 males and 10 females, all 12 days old) were placed in a cage to mate. Three days later, a 25 mL plastic cup containing 10 mL of commercially available orange juice was introduced as an oviposition site. The lid of the cup was modified to have 20 evenly spaced holes, each 1 mm in diameter, for egg laying. A 200-mesh gauze was placed between the lid and the orange juice to collect the eggs. The females were allowed approximately 10 hours to oviposit, and the number of eggs laid was manually counted. Additionally, 100 eggs were randomly selected to calculate their hatching rate. A total of 5 replicate were conducted.

Electrophysiological recording

Electroantennogram recordings: electroantennogram recordings (EAG) were performed using a modified version of the methodology previously reported by Xu et al. [10]. The procedure involved decapitating the male adult and severing one end of its antenna. Two glass electrodes were prepared, each filled with a 0.1M KCl solution. The reference electrode was attached to the severed head, and the recording electrode was connected to the cut end of the antenna. Recordings were taken from 12 adults of each genotype, specifically from unmated males that were 12 days old. EAG recordings were conducted using a BX 51 microscope (Olympus).

For air stimulation, the CS-55 controller (Syntech, Kirchzarten, Germany) was used. The airflow for stimulation was set at a rate of 1.4 ml/min, with a stimulation duration of 300 milliseconds. Signals were collected using a universal probe preamplifier and were then converted via a digital-to-analog converter (IDAC-4-USB, Syntech, Netherlands). The collected data were subsequently analyzed with EAGpro 2.0 software (Syntech, The Netherlands). The relative EAG responses were calculated by subtracting the baseline signal obtained with paraffin oil from the signal of the compound being tested.

Single-sensillum recording: single-sensillum recordings (SSR) were conducted using a modified version of the method previously reported by Liu et al. [11]. For *D. melanogaster* (fruit flies), the adult was secured in the head of a 10 μL pipette after removing the tip, allowing the head and antennae to protrude, and then fixed in place with dental wax. The recording electrode, made of tungsten, was inserted into the base of the sensillum, and the ab3A sensilla were identified by their response to the odors of 2-heptanone and ethyl acetate. The reference electrode was inserted into the compound eye of the fruit fly. We recorded from 5-6 fruit flies, with 10-14 sensilla in total, for the *22ab* ^{GAL4}*/UAS-BdorOR94b1* and its *GAL4* and *UAS* control strains. The response spectrum and dose-response to *BdorOR94b1* were recorded from 6 fruit flies, with 9-11 sensilla in total being recorded. For *B. dorsalis*, the males were fixed in a 200 μL pipette head after the tip was removed. The head was extended and secured with dental wax, and the antenna was affixed to a glass slide with double-sided adhesive tape. The recording electrode was inserted into the base of the sensillum, and the reference electrode was placed into the compound eye. The response spectrum and dose-response of ME receptors in wild-type males were recorded from 3-4 males, with 5-6 sensilla in total. When comparing *BdorOR94b1*$^{-/-}$ mutants with wild-type flies, recordings were taken from 3 wild-type males with 14 ME sensilla, and from 5 mutant males with 48 ME sensilla. SSR was performed using a BX51 microscope (Olympus).

Odor stimulation and signal acquisition were performed as described in the EAG method. The collected signals were processed using Autospike version 3.9 software. Low-frequency filtering was set at 300 Hz, while high-frequency filtering was set at 2 kHz. The response was quantified by counting the increase in the number of action potentials within 1 second following the stimulation.

Statistical analyses

All data are presented as the mean ± standard error. Initially, the normality of the original data was assessed using the Shapiro-Wilk test. If the data were not normally distributed, the Wilcoxon rank-sum test was used to compare differences between two groups, while the Kruskal-Wallis test was employed for three or more groups, followed by Dunn's multiple comparisons test. For normally distributed data, the two-tailed unpaired t-test was used for comparing differences between two groups, and one-way ANOVA was conducted for three or

more groups, followed by Tukey's multiple comparisons test. A P-value of less than 0.05 was considered to indicate statistical significance. All statistical analyses were performed using GraphPad Prism (Version 8.0.1).

REFERENCES

1. von der Weid B, Rossier D and Lindup M *et al*. Large-scale transcriptional profiling of chemosensory neurons identifies receptor-ligand pairs in vivo. *Nat Neurosci* 2015; **18**:1455–63.
2. Shen GM, Jiang HB and Wang XN *et al*. Evaluation of endogenous references for gene expression profiling in different tissues of the oriental fruit fly *Bactrocera dorsalis* (Diptera: Tephritidae). *BMC Mol Biol* 2010; **11**: 76.
3. Livak KJ and Schmittgen TD. Analysis of relative gene expression data using real-time quantitative PCR and the 2 (-Delta Delta C(T)) method. *Methods* 2001; **25**: 402–8.
4. Yuan JX Zhang J and Zhang Y *et al*. Protocols for CRISPR/Cas9 mutagenesis of the oriental fruit fly *Bactrocera dorsalis*. *J Vis Exp* 2022; **187**: No. e64195.
5. Xie SS, Shen B and Zhang CB *et al*. sgRNAcas9: a software package for designing CRISPR sgRNA and evaluating potential off-target cleavage sites. *PLoS One* 2014; **9**: e100448.
6. Ren C, Zhang J and Yuan JX *et al*. Light intensity regulates the sexual behaviors of oriental fruit fly *Bactrocera dorsalis* under laboratory conditions. *J Integr Agr* 2023; **22**: 2772–82.
7. Zhang Y, Wuyun QQG and Wang Q *et al*. MFS transporter *Bdorwp* does not affect antennal electrophysiology but regulates reproductive behaviors in *Bactrocera dorsalis*. *J Agric Food Chem* 2023; **71**: 17014–24.
8. Wuyun QQG, Zhang Y and Yuan JX *et al*. A classic screening marker does not affect antennal electrophysiology but strongly regulates reproductive behaviours in *Bactrocera dorsalis*. *Insect Mol Biol* 2023; **33**:136–46.
9. Roan CC, Flitters NE and Davis CJ. Light intensity and temperature as factors limiting the mating of the oriental fruit fly. *Ann Entomol Soc Am* 1954; **47**: 593–4.
10. Xu J, Liu W and Yang DH *et al*. Regulation of olfactory-based sex behaviors in the silkworm by genes in the sex-determination cascade. *PLoS Genet.* 2020; **16**: e1008622.
11. Liu W, Jiang XC and Cao S *et al*. Functional studies of sex pheromone receptors in Asian Corn Borer *Ostrinia furnacalis*. *Front Physiol* 2018; **9**: 591.

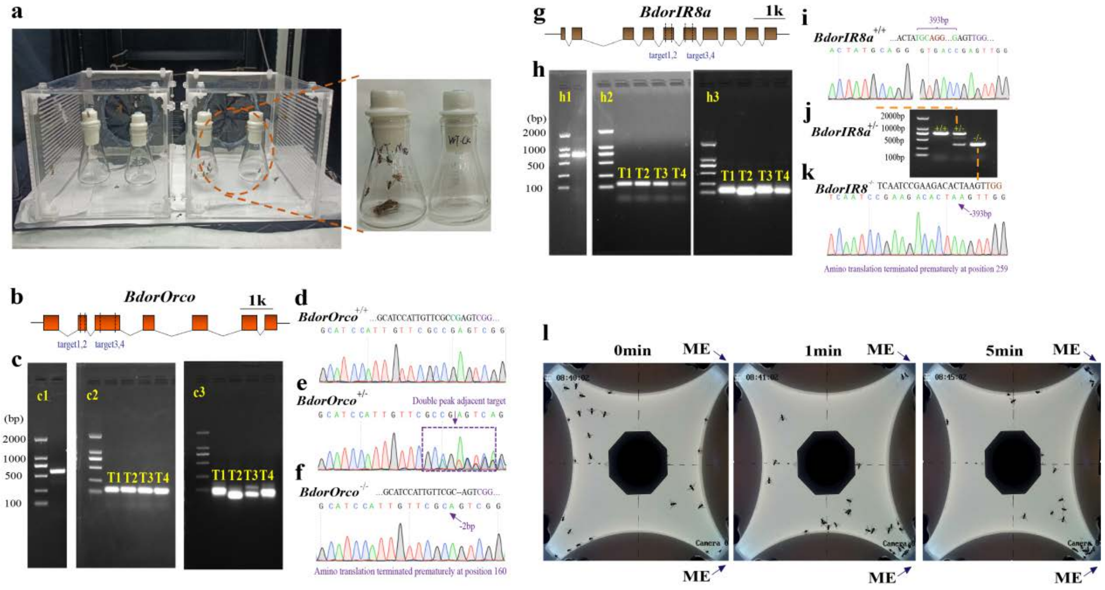

Figure. S1. Generation of *BdorOrco*$^{-/-}$ and *BdorIR8a*$^{-/-}$ mutants for behavioral testing. (a) Example of the olfactory trap assay. Traps were positioned inside an insect cage, with two traps per cage (left). Once inside, adult insects were unable to exit the trap (right). (b) Gene structure of the *BdorOrco* gene indicating the selected sgRNA target site. (c) Synthesis process of *BdorOrco* sgRNA. (d-f) Representative sequence chromatograms of PCR-amplified products from the target region of *BdorOrco*$^{+/+}$ (d), *BdorOrco*$^{+/-}$ (e), and *BdorOrco*$^{-/-}$ (f). (g) Gene structure of the *BdorIR8a* gene indicating the selected sgRNA target site. (h) Synthesis process of *BdorIR8a* sgRNAs. (i-k) Representative sequence chromatograms of PCR-amplified products from the target regions of *BdorIR8a*$^{+/+}$ (i), B*dorIR8a*$^{+/-}$ (j), and *BdorIR8a*$^{-/-}$ (k). (l) Examples of the four-quadrant olfactometer assay.

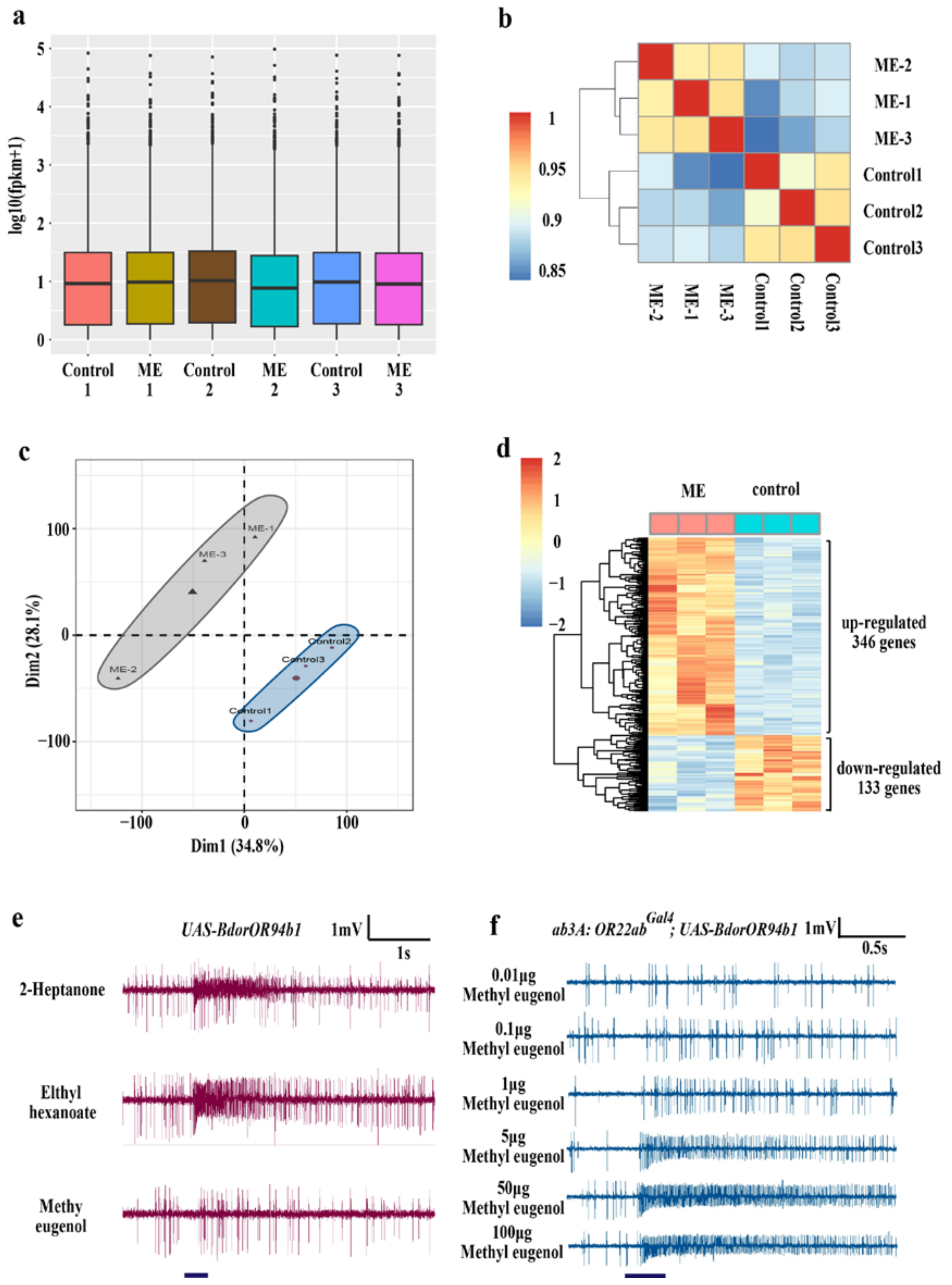

Figure S2. Screening olfactory receptor genes in response to ME. (a) Box plot illustrating the distribution of FPKM values for gene expression levels across all RNA samples. (b) Heatmap depicting Pearson correlation analysis among all RNA samples. The color scale represents the Pearson correlation coefficient, with red indicating high correlation and blue indicating low correlation. (c) Principal Component Analysis (PCA) demonstrating the variance between the ME-treated group and the control group. (d) Heatmap displaying genes that are differentially expressed between ME-treated and control samples. Rows represent genes with significant

expression differences between the two groups, while columns correspond to individual samples from each group. The color scale indicates gene expression levels, with red representing increased expression and blue representing decreased expression. (e) Representative SSR traces from ab3A sensilla of *UAS-BdorOR94b1 Drosophila* in response to 2-heptanone, ethyl hexanoate, and ME. (f) Representative SSR traces of ab3A sensilla responses to different doses of ME in *OR22ab*^{Gal4} > *UAS-BdorOR94b1 Drosophila*.

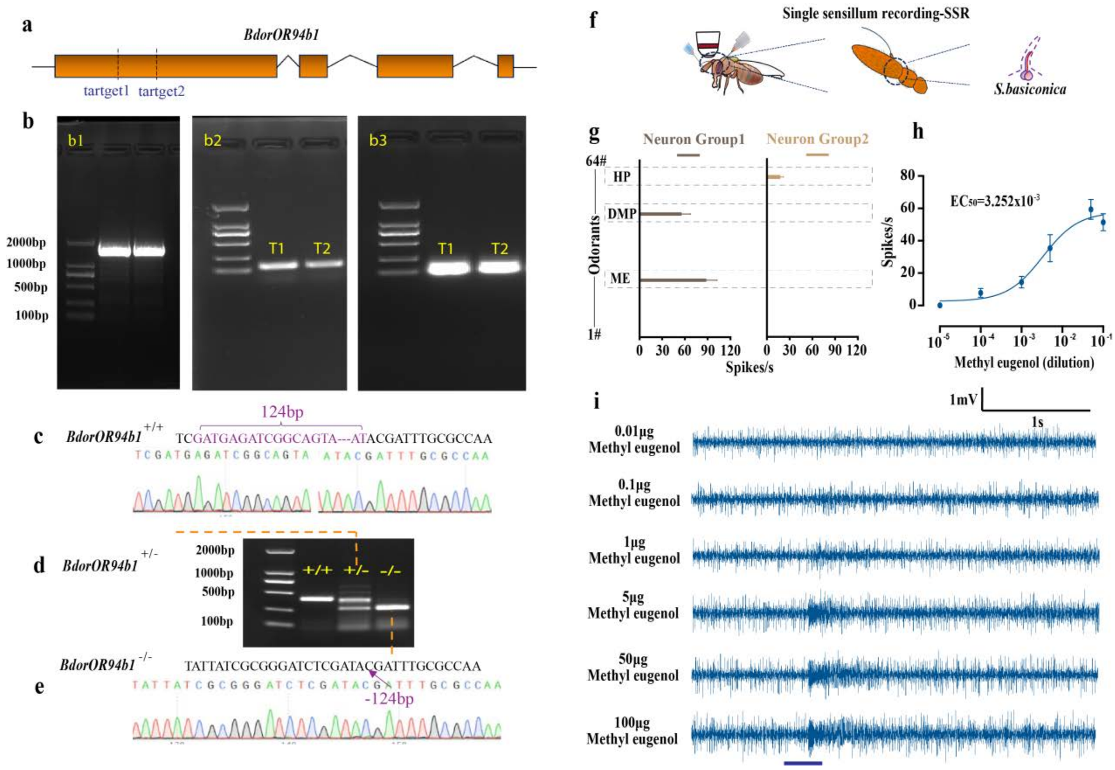

Figure S3. Knockout of *BdorOR94b1* abolishes the electrophysiological response of *B. dorsalis* to ME. (a) Gene structure of the *BdorOR94b1* gene, indicating the selected sgRNA target site. (b) Synthesis process of *BdorOR94b1* sgRNA. (c-e) Representative sequence chromatograms of PCR-amplified products from the target region of *BdorOR94b1*$^{+/+}$ (c), *BdorOR94b1*$^{+/-}$ (d), and *BdorOR94b1*$^{-/-}$ (e). (f) Schematic drawing of SSR experiments in *B. dorsalis* males. (g) Quantification of SSR responses to 64 different odorants (listed in Table S3) by *s. basiconica* in WT males. Neurons were categorized into Group 1 and Group 2 based on their response characteristics. (h) SSR dose-response curve of *BdorOR94b1* neurons to ME. Data are presented as mean ± standard error, N=6 sensilla across 4 individuals. (i) Representative SSR traces from *s. basiconica* in males stimulated with different concentrations of ME.

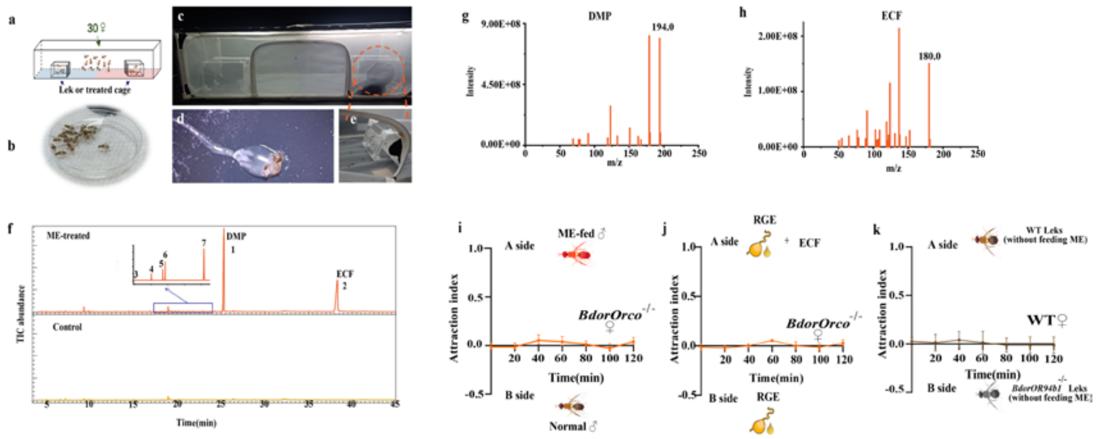

Figure S4. ME aids female locating male leks by converting into two prominent components, ECF, and 2-allyl-4,5-dimethoxyphenol (DMP) in male rectal glands. (a) Schematic of dual-choice lek experiments. (b) Representative image showing male feeding behavior towards ME. (c) Representative setup used in dual-choice lek experiments. (d) Dissection of the male rectal gland under a stereomicroscope. (e) Females attracted to the artificial leks during the preference experiment. (f) Gas chromatograms of male rectal gland extraction with or without ME feeding. (g-h) Total ion GC-MS chromatograms of DMP and ECF. (i) Behavioral choice of *BdorOrco*$^{-/-}$ female oriental flies between leks containing ME-fed or untreated males. (j) Behavioral choice of BdorOrco$^{-/-}$ female oriental flies between leks supplemented with untreated male rectal gland extract plus ECF versus leks with pure gland extract. (k) Behavioral choice of WT female oriental flies between leks containing WT and *BdorOR94b1*$^{-/-}$ males.

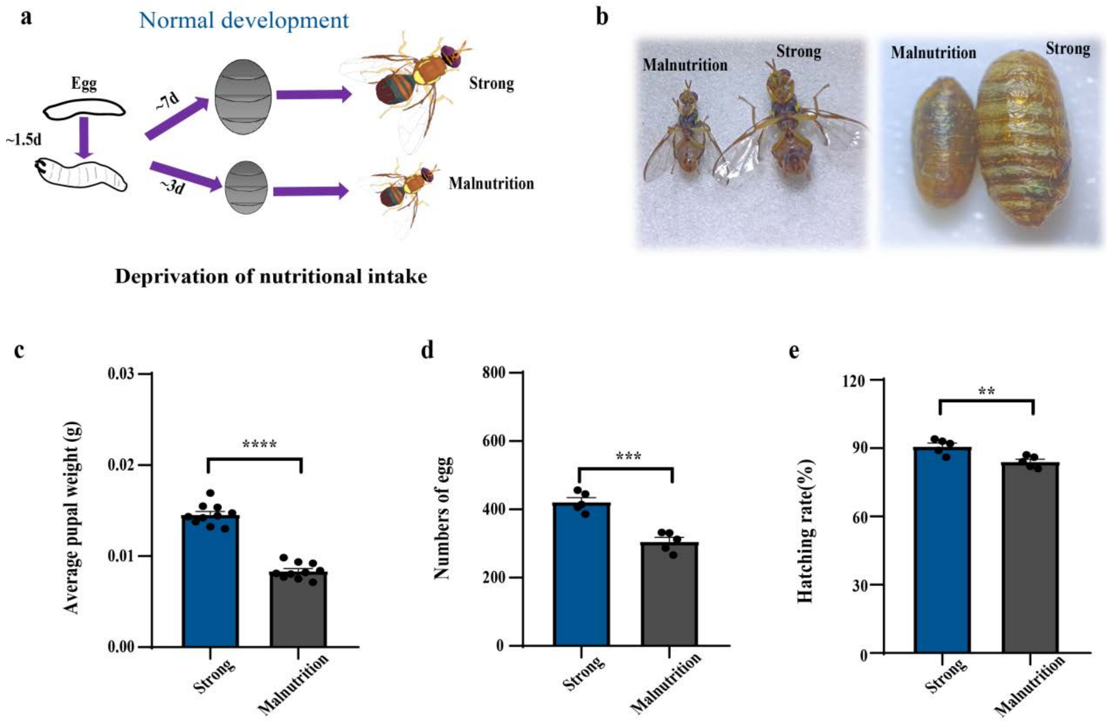

Figure S5. Effects of nutritional deprivation on body size, pupal weight, egg production, and hatch rates of *B. dorsalis*. (a) Experimental design for malnutrition treatment of *B. dorsalis* larvae. (b) Malnutrition treatment results in reduced pupal and adult size. (c) Comparison of pupal weights between normally fed and malnutrition-treated groups. (d) Comparison of fecundity between adults from normal and malnutrition treatments. (e) Comparison of hatch rates of offspring eggs from adults subjected to normal and malnutrition treatments. In all experiments, N=5, and data are presented as mean ± standard error. A two-tailed unpaired t-test was used to analyze differences in the data (**$p < 0.01$, ***$p < 0.001$, ****$p < 0.0001$).
